# Supplementary material for: Neurofilaments in spinocerebellar ataxia type 3: blood biomarkers at the preataxic and ataxic stage in humans and mice
Source: EMBO Mol Med. 2020 Jun 8;12(7):e11803. doi: 10.15252/emmm.201911803 (PMC7338806; doi:10.15252/emmm.201911803)
Supplement: Supplementary file 1 — Appendix [file EMMM-12-e11803-s001.pdf]

Appendix

Table of Contents

Appendix Table S1.	Neuropathologic assessment of degeneration and polyQ pathology in a SCA3 subject with increased Nf levels.
Appendix Table S2.	Analytical characteristics and validation of the neurofilament assays.
Appendix Table S3.	Statistical details of figures.
Appendix Table S4.	SCA3 Neurofilament Study Group.
Appendix Figure S1.	Weight phenotype in the 304Q knock-in SCA3 mouse model.
Appendix Figure S2.	Motor phenotype in the 304Q knock-in SCA3 mouse model.
Appendix Figure S3.	Quantitative assessment of Purkinje cell alterations and cell loss in the 304Q SCA3 mouse model.
Appendix Figure S4.	Quantitative assessment of general atrophy in the 304Q SCA3 mouse model.

Appendix Supplementary References.

Appendix Table S1. Neuropathologic assessment of degeneration and polyQ pathology in a SCA3 subject with increased Nf levels.

Methods. Post-mortem tissue of an ataxic SCA3 subject (ID27925), for whom also Nf levels were available, was provided by the brain bank affiliated with the University Hospital of Tübingen. Clinical assessment of subject ID27925 at the age of 48.5 years (ataxia onset: 34 years, disease duration: 15 years, death: 49.5 years, repeat length: 72) showed severe clinical affection (SARA score: 32) and above-average disease progression (2.2 SARA points/year; average progression in SCA3: 1.56 SARA points/year (Jacobi *et al*, 2015)). The subject showed serum NfL levels of 116 pg/ml (93. percentile within the ataxic subjects of cohort #2) and serum pNfH levels of 23.2 pg/ml (52. percentile). Histological examination was performed on 4 µm thick sections cut from formalin-fixed, paraffin-embedded tissue of frontal cortex, temporal cortex, primary motor cortex, putamen/pallidum, midbrain, pons, medulla, cerebellum and spinal cord. Sections were stained with haematoxylin and eosin (H&E), and Luxol fast blue–periodic acid–Schiff, or used for immunohistochemistry using the Ventana BenchMark XT automated staining system with the OptiView DAB detection kit (Ventana). Antibodies employed include monoclonal mouse anti-CD68 clone PG-M1 (Dako) and anti-polyQ clone 1C2 (Millipore). A semiquantitative grading system was used to score the severity of polyQ pathology (neuronal nuclear inclusions and granular cytoplasmic staining) as absent, mild (only few inclusions seen in the entire region examined), moderate (at least a few inclusions present in most microscopic fields) or severe (many inclusions in every microscopic field). Degeneration of brain regions and fibre tracts was assessed on H&E, myelin and CD68 stained sections and graded as absent, mild, moderate, severe based on the presence of spongiosis, neuronal loss, gliosis and/or myelin loss.

Results. Severity and distribution of degeneration and polyQ pathology are summarised in the table below (semiquantitative score: - absent, + mild, ++ moderate, +++ severe, NA: not applicable). The neuropathological findings indicate, in sum, that the spinocerebellar tract, brainstem and basal ganglia, but not the corticospinal tract, motor cortex or cerebellar cortex were mainly affected in this subject. These findings, which correspond with and confirm the findings of previous SCA3 neuropathology case series (Koeppen, 2018; Paulson, 2012; Paulson *et al*, 2017), might provide first preliminary indications of the central nervous system regions underlying the Nf increase in SCA3.

CNS region	degeneration	polyQ pathology
**cerebellum**		
Purkinje cells	+	-
dentate nucleus	++	+++
**brainstem**		
pontine nuclei	++	+++
substantia nigra pars compacta	++	++
oculomotor nuclei	++	++
inferior olive	+	+++
XII nucleus	+	+++
**cortex**		
basal ganglia	+	+++
primary motor cortex (layer V giant Betz cells)	+	+
frontal	-	+
temporal	-	+
**spinal cord**		
anterior horn	++	+++
**fibre tracts**		
corticospinal tracts	+	NA
spinocerebellar tracts	++	NA
pontocerebellar fibres	++	NA
cerebellar peduncles	++	NA

Appendix Table S2. Analytical characteristics and validation of the neurofilament assays.

In line with previous studies (Kuhle *et al*, 2016; Wilke *et al*, 2019), analytical sensitivity of each assay was defined as the analyte concentration of the calibrator with the lowest concentration fulfilling established acceptance criteria (i.e. coefficient of variation (CV) of duplicate determination ≤20% and accuracy within the range of 80-120%). Analytical sensitivity was corrected by multiplication with the dilution factor (i.e. multiplication with factor 4). Within-run precision and between-run precision were derived from four consecutive runs with samples of different analyte concentrations. Sample CVs were based on duplicate measurements of all available samples. Data were reported as median and interquartile range, unless stated otherwise.

**analyte**	**NfL**		**pNfH**	
**assay**	**homebrew Simoa**	**Quanterix Simoa**	**homebrew Simoa**	**Quanterix Simoa**
**analytical sensitivity**	2.4 pg/ml	1.6 pg/ml	3.2 pg/ml	11.2 pg/ml
**within-run precision**	7.4% (30.5 pg/ml)<br>7.6% (92.7 pg/ml)<br>6.2% (491.6 pg/ml)	4.8% (10.1 pg/ml)<br>11.3% (51.6 pg/ml)<br>12.3% (155.1 pg/ml)	2.9% (32.1 pg/ml)<br>4.9% (19.8 pg/ml)<br>6.2% (89.7 pg/ml)	5.4% (18.5 pg/ml)<br>5.2% (267.1 pg/ml)<br>7.9% (1664 pg/ml)
**between-run precision**	9.5% (30.5 pg/ml)<br>11.1% (92.7 pg/ml)<br>8.9% (491.6 pg/ml)	7.2% (10.1 pg/ml)<br>11.3% (51.6 pg/ml)<br>15.4% (155.1 pg/ml)	6.2% (32.1 pg/ml)<br>6.8% (19.8 pg/ml)<br>13.1% (89.7 pg/ml)	12.1% (18.5 pg/ml)<br>7.5% (267.1 pg/ml)<br>9.9% (1664 pg/ml)
**sample CV**	3.2% (1.1-5.4)	4.1% (1.7-7.3)	5.7% (3.1-11.0)	4.2% (1.6-7.0)

Assay validation in 47 independent serum samples (13 SCA3 subjects, 34 controls) demonstrated high agreement between the Quanterix and the homebrew NfL assays (R=0.99) (A). This allowed transformation of the NfL homebrew measurements to the scale of the Quanterix measurements by linear regression of log-transformed values and consecutive pooling of NfL values across the two cohorts. For pNfH, analogous validation showed lesser agreement between the Quanterix and the homebrew assays (R=0.88) (B), which we considered insufficient for transformation of pNfH homebrew measurements to the scale of the Quanterix measurements.

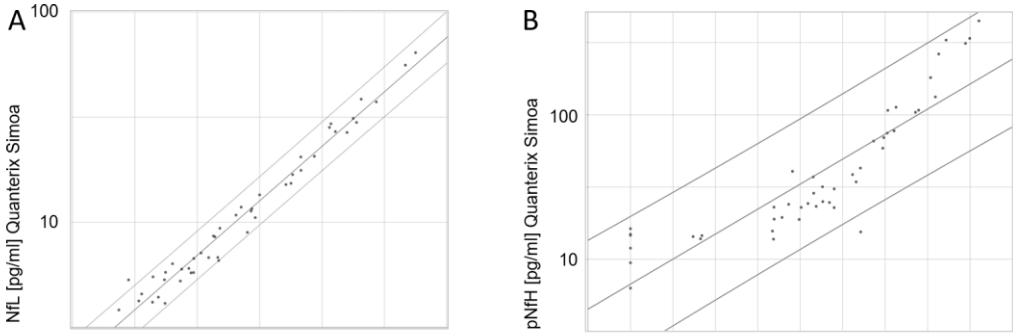

Appendix Table S3. Statistical details of figures.

figure	analysis	statistic
1A	NfL, ataxic vs. controls	U = 151, z = 10.1, p = 2.57E-33
	NfL, preataxic vs. controls	U = 72, z = 3.55, p = 0.00011
	NfL, ataxic vs. preataxic	U = 204, z = 1.48, p = 0.143
1B	NfL, ataxic vs. controls	U = 16, z = 6.98, p = 9.97E-18
	NfL, preataxic vs. controls	U = 88, z = 4.18, p = 0.00001
	NfL, ataxic vs. preataxic	U = 74, z = 3.16, p = 0.00112
1E	pNfH, ataxic vs. controls	U = 1064, z = 6.72, p = 1.26E-12
	pNfH, preataxic vs. controls	U = 201, z = 1.61, p = 0.109
	pNfH, ataxic vs. preataxic	U = 186, z = 1.76, p = 0.080
1F	pNfH, ataxic vs. controls	U = 260, z = 4.57, p = 3.19E-7
	pNfH, preataxic vs. controls	U = 250, z = 1.63, p = 0.103
	pNfH, ataxic vs. preataxic	U = 109, z = 2.20, p = 0.084
2A	correlation of NfL with SARA score	r = 0.43, p = 0.00025
2B	NfL, low vs. high progression disease progression	U = 82, z = 2.34, p = 0.018
5A	NfL, plasma, wildtype vs. heterozygous, 2 months	t(19) = -2.21, p = 0.080
	NfL, plasma, wildtype vs. heterozygous, 6 months	t(30) = 4.81, p = 0.00008
	NfL, plasma, wildtype vs. heterozygous, 12 months	t(16.53) = 4.96, p = 0.00026
	NfL, plasma, wildtype vs. heterozygous, 18 months	t(26) = 0.95, p = 0.704
5B	pNfH, plasma, wildtype vs. heterozygous, 2 months	t(20) = 0.00, p = 0.999
	pNfH, plasma, wildtype vs. heterozygous, 6 months	t(16.61) = 4.00, p = 0.002
	pNfH, plasma, wildtype vs. heterozygous, 12 months	t(14.35) = 5.23, p = 0.00023
	pNfH, plasma, wildtype vs. heterozygous, 18 months	t(27) = -0.36, p = 0.999
5C	soluble ataxin-3, cerebellar, wildtype vs. heterozygous, 2 months	t(19) = 8.78, p < 0.00001
	soluble ataxin-3, cerebellar, wildtype vs. heterozygous, 6 months	t(30) = 5.58, p = 0.00001
	soluble ataxin-3, cerebellar, wildtype vs. heterozygous, 12 months	t(25) = 4.32, p = 0.00043
	soluble ataxin-3, cerebellar, wildtype vs. heterozygous, 18 months	t(25.13) = 4.62, p = 0.00020
5D	soluble ataxin-3, frontal, wildtype vs. heterozygous, 2 months	t(19) = 3.11, p = 0.012
	soluble ataxin-3, frontal, wildtype vs. heterozygous, 6 months	t(29) = 6.49, p < 0.00001
	soluble ataxin-3, frontal, wildtype vs. heterozygous, 12 months	t(25) = 4.51, p = 0.00027
	soluble ataxin-3, frontal, wildtype vs. heterozygous, 18 months	t(26) = 3.18, p = 0.008
5E	ataxin-3 aggregated, cerebellar, wildtype vs. heterozygous, 2 months	t(4) = 3.16, p = 0.034
	ataxin-3 aggregated, cerebellar, wildtype vs. heterozygous, 6 months	t(4) = 2.04, p = 0.111
	ataxin-3 aggregated, cerebellar, wildtype vs. heterozygous, 12 months	t(4) = 1.98, p = 0.118

figure	analysis	statistic
5E	ataxin-3 aggregated, cerebellar, wildtype vs. heterozygous, 18 months	t(4) = 3.35, p = 0.029
5F	ataxin-3 aggregated, frontal, wildtype vs. heterozygous, 2 months	t(4) = 2.69, p = 0.055
	ataxin-3 aggregated, frontal, wildtype vs. heterozygous, 6 months	t(2.21) = 2.93, p = 0.088
	ataxin-3 aggregated, frontal, wildtype vs. heterozygous, 12 months	t(4) = 16.53, p = 0.00008
	ataxin-3 aggregated, frontal, wildtype vs. heterozygous, 18 months	t(4) = 7.46, p = 0.002
EV2A	NfL, plasma, wildtype vs. heterozygous, 2 months	t(19) = -2.21, p = 0.080
	NfL, plasma, wildtype vs. heterozygous, 6 months	t(30) = 4.81, p = 0.00008
	NfL, plasma, wildtype vs. heterozygous, 12 months	t(16.53) = 4.96, p = 0.00026
	NfL, plasma, wildtype vs. heterozygous, 18 months	t(26) = 0.95, p = 0.704
	NfL, plasma, wildtype vs. homozygous, 2 months	t(25) = -0.35, p = 0.999
	NfL, plasma, wildtype vs. homozygous, 6 months	t(24) = 6.45, p < 0.00001
	NfL, plasma, wildtype vs. homozygous, 12 months	t(20) = 3.20, p = 0.009
	NfL, plasma, wildtype vs. homozygous, 18 months	t(12) = -0.65, p = 0.999
EV2B	pNfH, plasma, wildtype vs. heterozygous, 2 months	t(20) = 0.00, p = 0.999
	pNfH, plasma, wildtype vs. heterozygous, 6 months	t(16.61) = 4.00, p = 0.002
	pNfH, plasma, wildtype vs. heterozygous, 12 months	t(14.35) = 5.23, p = 0.00023
	pNfH, plasma, wildtype vs. heterozygous, 18 months	t(27) = -0.36, p = 0.999
	pNfH, plasma, wildtype vs. homozygous, 2 months	t(26) = 0.00, p = 0.999
	pNfH, plasma, wildtype vs. homozygous, 6 months	t(10.35) = 4.14, p = 0.004
	pNfH, plasma, wildtype vs. homozygous, 12 months	t(9.13) = 3.32, p = 0.018
	pNfH, plasma, wildtype vs. homozygous, 18 months	t(13) = 0.27, p = 0.999
EV2C	NfL, plasma, female, wildtype vs. heterozygous, 2 months	t(9) = -2.17, p = 0.116
	NfL, plasma, female, wildtype vs. heterozygous, 6 months	t(14) = 5.88, p = 0.00008
	NfL, plasma, female, wildtype vs. heterozygous, 12 months	t(10) = 3.51, p = 0.011
	NfL, plasma, female, wildtype vs. heterozygous, 18 months	t(16) = 0.56, p = 0.999
	NfL, plasma, female, wildtype vs. homozygous, 2 months	t(12) = -0.67, p = 0.999
	NfL, plasma, female, wildtype vs. homozygous, 6 months	t(8.97) = 8.06, p = 0.00004
	NfL, plasma, female, wildtype vs. homozygous, 12 months	t(7) = 3.98, p = 0.011
	NfL, plasma, female, wildtype vs. homozygous, 18 months	t(6) = -0.26, p = 0.999
EV2D	pNfH, plasma, female, wildtype vs. heterozygous, 2 months	t(9) = 0.00, p = 0.999
	pNfH, plasma, female, wildtype vs. heterozygous, 6 months	t(8.30) = 2.79, p = 0.045
	pNfH, plasma, female, wildtype vs. heterozygous, 12 months	t(6.37) = 3.25, p = 0.032
	pNfH, plasma, female, wildtype vs. heterozygous, 18 months	t(16) = -0.82, p = 0.849
	pNfH, plasma, female, wildtype vs. homozygous, 2 months	t(12) = 0.00, p = 0.999
	pNfH, plasma, female, wildtype vs. homozygous, 6 months	t(10) = 6.23, p = 0.00019

figure	analysis	statistic
EV2D	pNfH, plasma, female, wildtype vs. homozygous, 12 months	t(3.06) = 2.69, p = 0.145
	pNfH, plasma, female, wildtype vs. homozygous, 18 months	t(6) = -0.24, p = 0.999
EV2E	NfL, plasma, male, wildtype vs. heterozygous, 2 months	t(8) = -1.53, p = 0.331
	NfL, plasma, male, wildtype vs. heterozygous, 6 months	t(14) = 1.86, p = 0.169
	NfL, plasma, male, wildtype vs. heterozygous, 12 months	t(13) = 3.85, p = 0.004
	NfL, plasma, male, wildtype vs. heterozygous, 18 months	t(8) = 0.69, p = 0.999
	NfL, plasma, male, wildtype vs. homozygous, 2 months	t(11) = 0.78, p = 0.909
	NfL, plasma, male, wildtype vs. homozygous, 6 months	t(12) = 4.11, p = 0.003
	NfL, plasma, male, wildtype vs. homozygous, 12 months	t(7.51) = 1.42, p = 0.394
	NfL, plasma, male, wildtype vs. homozygous, 18 months	t(4) = -0.69, p = 0.999
EV2F	pNfH, plasma, male, wildtype vs. heterozygous, 2 months	t(9) = 0.00, p = 0.999
	pNfH, plasma, male, wildtype vs. heterozygous, 6 months	t(7.38) = 2.97, p = 0.039
	pNfH, plasma, male, wildtype vs. heterozygous, 12 months	t(13) = 3.57, p = 0.007
	pNfH, plasma, male, wildtype vs. heterozygous, 18 months	t(9) = -0.21, p = 0.999
	pNfH, plasma, male, wildtype vs. homozygous, 2 months	t(12) = 0.00, p = 0.999
	pNfH, plasma, male, wildtype vs. homozygous, 6 months	t(5.10) = 2.10, p = 0.178
	pNfH, plasma, male, wildtype vs. homozygous, 12 months	t(5) = 3.33, p = 0.042
	pNfH, plasma, male, wildtype vs. homozygous, 18 months	t(5) = 0.61, p = 0.999
EV3A	NfL, cerebellar, wildtype vs. heterozygous, 2 months	t(20) = -1.44, p = 0.330
	NfL, cerebellar, wildtype vs. heterozygous, 6 months	t(30) = 2.32, p = 0.054
	NfL, cerebellar, wildtype vs. heterozygous, 12 months	t(25) = -0.50, p = 0.999
	NfL, cerebellar, wildtype vs. heterozygous, 18 months	t(27) = 2.54, p = 0.035
	NfL, cerebellar, wildtype vs. homozygous, 2 months	t(26) = -2.08, p = 0.094
	NfL, cerebellar, wildtype vs. homozygous, 6 months	t(24) = 1.42, p = 0.339
	NfL, cerebellar, wildtype vs. homozygous, 12 months	t(20) = -1.20, p = 0.487
	NfL, cerebellar, wildtype vs. homozygous, 18 months	t(13) = 0.07, p = 0.999
EV3B	NfL, frontal, wildtype vs. heterozygous, 2 months	t(19) = -1.21, p = 0.484
	NfL, frontal, wildtype vs. heterozygous, 6 months	t(30) = 0.76, p = 0.902
	NfL, frontal, wildtype vs. heterozygous, 12 months	t(25) = -1.10, p = 0.565
	NfL, frontal, wildtype vs. heterozygous, 18 months	t(27) = -0.87, p = 0.788
	NfL, frontal, wildtype vs. homozygous, 2 months	t(26) = -0.58, p = 0.999
	NfL, frontal, wildtype vs. homozygous, 6 months	t(23) = -0.95, p = 0.708
	NfL, frontal, wildtype vs. homozygous, 12 months	t(20) = -2.02, p = 0.114
	NfL, frontal, wildtype vs. homozygous, 18 months	t(12) = -2.31, p = 0.079
EV3C	pNfH, cerebellar, wildtype vs. heterozygous, 2 months	t(20) = -0.33, p = 0.999

figure	analysis	statistic
EV3C	pNfH, cerebellar, wildtype vs. heterozygous, 6 months	$t(30) = 1.73$, $p = 0.186$
	pNfH, cerebellar, wildtype vs. heterozygous, 12 months	$t(25) = 1.18$, $p = 0.499$
	pNfH, cerebellar, wildtype vs. heterozygous, 18 months	$t(27) = 1.80$, $p = 0.167$
	pNfH, cerebellar, wildtype vs. homozygous, 2 months	$t(26) = -1.36$, $p = 0.370$
	pNfH, cerebellar, wildtype vs. homozygous, 6 months	$t(24) = 0.43$, $p = 0.999$
	pNfH, cerebellar, wildtype vs. homozygous, 12 months	$t(20) = 2.06$, $p = 0.104$
	pNfH, cerebellar, wildtype vs. homozygous, 18 months	$t(13) = 0.64$, $p = 0.999$
EV3D	pNfH, frontal, wildtype vs. heterozygous, 2 months	$t(19) = -0.86$, $p = 0.800$
	pNfH, frontal, wildtype vs. heterozygous, 6 months	$t(30) = 1.33$, $p = 0.388$
	pNfH, frontal, wildtype vs. heterozygous, 12 months	$t(25) = 0.35$, $p = 0.999$
	pNfH, frontal, wildtype vs. heterozygous, 18 months	$t(27) = 0.10$, $p = 0.999$
	pNfH, frontal, wildtype vs. homozygous, 2 months	$t(26) = 0.19$, $p = 0.999$
	pNfH, frontal, wildtype vs. homozygous, 6 months	$t(23) = 0.71$, $p = 0.974$
	pNfH, frontal, wildtype vs. homozygous, 12 months	$t(20) = 0.48$, $p = 0.999$
	pNfH, frontal, wildtype vs. homozygous, 18 months	$t(12) = -2.06$, $p = 0.123$
EV4A	soluble ataxin-3, cerebellar, wildtype vs. heterozygous, 2 months	$t(19) = 8.78$, $p < 0.00001$
	soluble ataxin-3, cerebellar, wildtype vs. heterozygous, 6 months	$t(30) = 5.58$, $p = 0.00001$
	soluble ataxin-3, cerebellar, wildtype vs. heterozygous, 12 months	$t(25) = 4.32$, $p = 0.00043$
	soluble ataxin-3, cerebellar, wildtype vs. heterozygous, 18 months	$t(25.13) = 4.62$, $p = 0.00020$
	soluble ataxin-3, cerebellar, wildtype vs. homozygous, 2 months	$t(18.41) = 8.05$, $p < 0.00001$
	soluble ataxin-3, cerebellar, wildtype vs. homozygous, 6 months	$t(24) = 8.04$, $p < 0.00001$
	soluble ataxin-3, cerebellar, wildtype vs. homozygous, 12 months	$t(20) = 4.32$, $p = 0.00067$
	soluble ataxin-3, cerebellar, wildtype vs. homozygous, 18 months	$t(12) = 2.01$, $p = 0.134$
EV4B	soluble ataxin-3, frontal, wildtype vs. heterozygous, 2 months	$t(19) = 3.11$, $p = 0.012$
	soluble ataxin-3, frontal, wildtype vs. heterozygous, 6 months	$t(29) = 6.49$, $p < 0.00001$
	soluble ataxin-3, frontal, wildtype vs. heterozygous, 12 months	$t(25) = 4.51$, $p = 0.00027$
	soluble ataxin-3, frontal, wildtype vs. heterozygous, 18 months	$t(26) = 3.18$, $p = 0.008$
	soluble ataxin-3, frontal, wildtype vs. homozygous, 2 months	$t(25) = 11.90$, $p < 0.00001$
	soluble ataxin-3, frontal, wildtype vs. homozygous, 6 months	$t(23) = 5.94$, $p = 0.00001$
	soluble ataxin-3, frontal, wildtype vs. homozygous, 12 months	$t(15.37) = 6.63$, $p = 0.00001$
	soluble ataxin-3, frontal, wildtype vs. homozygous, 18 months	$t(12) = 2.08$, $p = 0.118$
EV4C	ataxin-3 aggregated, cerebellar, wildtype vs. heterozygous, 2 months	$t(4) = 3.16$, $p = 0.034$
	ataxin-3 aggregated, cerebellar, wildtype vs. heterozygous, 6 months	$t(4) = 2.04$, $p = 0.111$
	ataxin-3 aggregated, cerebellar, wildtype vs. heterozygous, 12 months	$t(4) = 1.98$, $p = 0.118$
	ataxin-3 aggregated, cerebellar, wildtype vs. heterozygous, 18 months	$t(4) = 3.35$, $p = 0.029$

figure	analysis	statistic
EV4C	ataxin-3 aggregated, cerebellar, wildtype vs. homozygous, 2 months	$t(4) = 3.12$, $p = 0.036$
	ataxin-3 aggregated, cerebellar, wildtype vs. homozygous, 6 months	$t(4) = 7.05$, $p = 0.002$
	ataxin-3 aggregated, cerebellar, wildtype vs. homozygous, 12 months	$t(4) = 4.28$, $p = 0.013$
	ataxin-3 aggregated, cerebellar, wildtype vs. homozygous, 18 months	$t(4) = 5.69$, $p = 0.005$
EV4D	ataxin-3 aggregated, frontal, wildtype vs. heterozygous, 2 months	$t(4) = 2.69$, $p = 0.055$
	ataxin-3 aggregated, frontal, wildtype vs. heterozygous, 6 months	$t(2.21) = 2.93$, $p = 0.088$
	ataxin-3 aggregated, frontal, wildtype vs. heterozygous, 12 months	$t(4) = 16.53$, $p = 0.00008$
	ataxin-3 aggregated, frontal, wildtype vs. heterozygous, 18 months	$t(4) = 7.46$, $p = 0.002$
	ataxin-3 aggregated, frontal, wildtype vs. homozygous, 2 months	$t(4) = 3.90$, $p = 0.018$
	ataxin-3 aggregated, frontal, wildtype vs. homozygous, 6 months	$t(4) = 5.73$, $p = 0.005$
	ataxin-3 aggregated, frontal, wildtype vs. homozygous, 12 months	$t(4) = 8.74$, $p = 0.00095$
	ataxin-3 aggregated, frontal, wildtype vs. homozygous, 18 months	$t(2.02) = 12.74$, $p = 0.006$

Appendix Table S4. SCA3 Neurofilament Study Group.

**author**	**affiliation**
Christian Deuschle	Hertie Institute for Clinical Brain Research (HIH) and Center of Neurology, University of Tübingen, Germany.<br>German Center for Neurodegenerative Diseases (DZNE), University of Tübingen, Germany.
Elke Stransky	Hertie Institute for Clinical Brain Research (HIH) and Center of Neurology, University of Tübingen, Germany.
Kathrin Brockmann	Hertie Institute for Clinical Brain Research (HIH) and Center of Neurology, University of Tübingen, Germany.<br>German Center for Neurodegenerative Diseases (DZNE), University of Tübingen, Germany.
Jörg B. Schulz	Department of Neurology, RWTH Aachen University, Aachen, Germany.<br>JARA-BRAIN Institute Molecular Neuroscience and Neuroimaging, Forschungszentrum Jülich, RWTH Aachen University, Aachen, Germany.
Laszlo Baliko	Department of Medical Genetics, and Szentagothai Research Center, University of Pécs Medical School, Pécs, Hungary.
Judith van Gaalen	Donders Institute for Brain, Cognition, and Behaviour, Department of Neurology, Radboud university medical center, Nijmegen, The Netherlands.
Mafalda Raposo	Faculdade de Ciências e Tecnologia, Universidade dos Açores, Ponta Delgada, Portugal.
Andreas Jeromin	Quanterix Corporation, Lexington, USA.

Appendix Figure S1. Weight phenotype in the 304Q knock-in SCA3 mouse model.

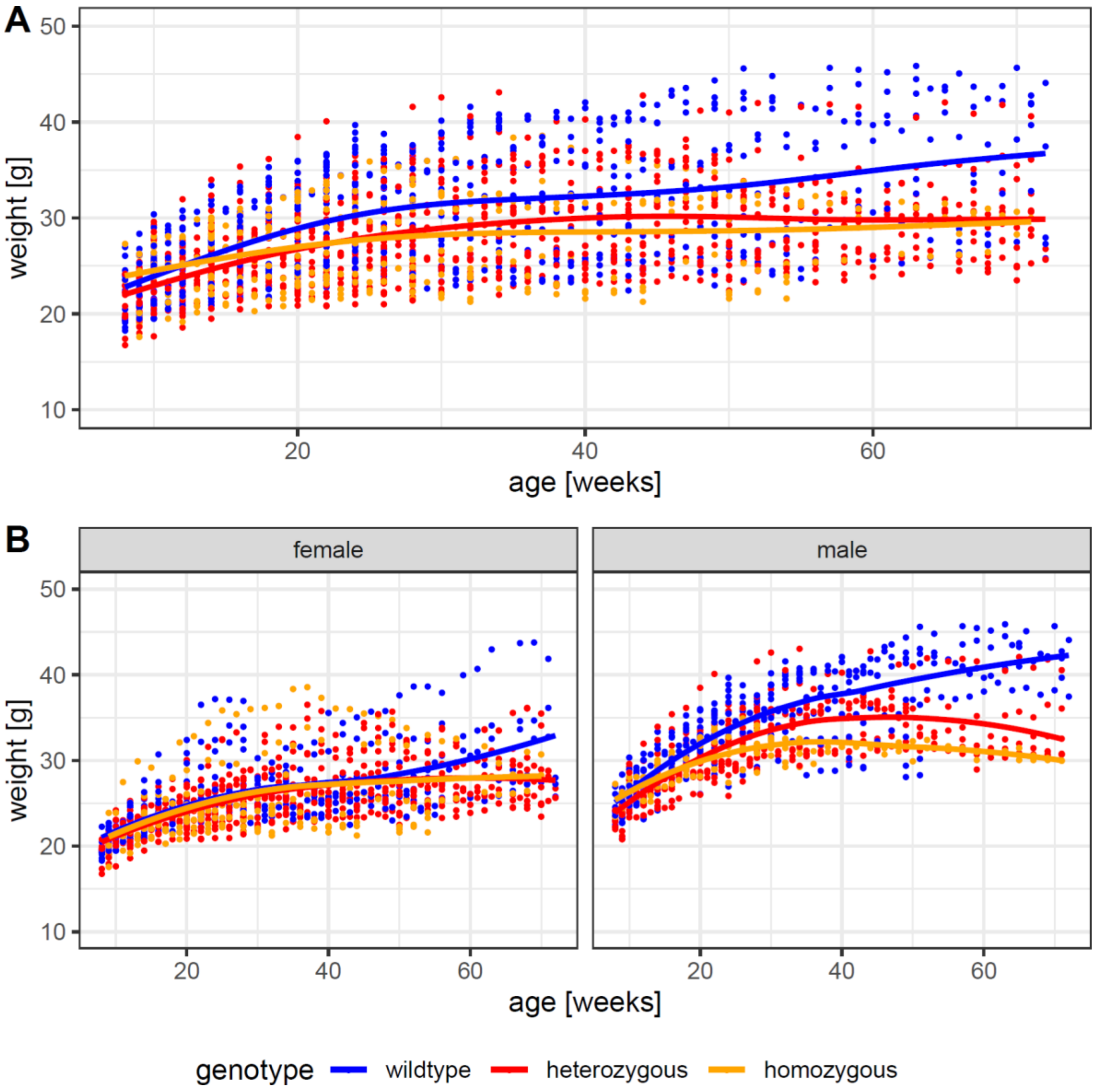

Weight was determined in all animals (n=145) every two weeks. For each genotype, the mean was estimated by LOESS technique (locally estimated scatterplot smoothing, with standard span, as implemented in the R package ggplot2). With both sexes pooled **(A)**, heterozygous animals started differing from wildtype animals in weight at age 12 months. Analysing both sexes separately **(B)**, we observed the onset of the weight phenotype for heterozygous animals at age 7 months in males and 12 months in females.

Appendix Figure S2. Motor phenotype in the 304Q knock-in SCA3 mouse model.

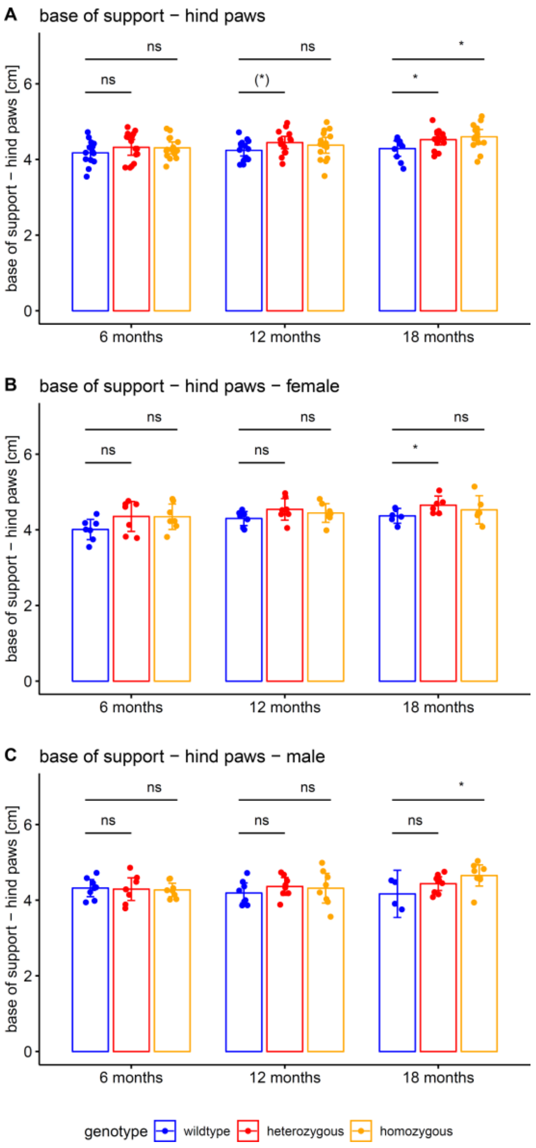

The motor phenotype of the 304Q knock-in SCA3 mouse model was assessed by automated gait analysis in the Catwalk system. Balance was captured by the base of support of the animal's hind paws. Hetero- and homozygous animals were compared to wildtype animals by two-tailed unpaired t tests (* p<.05, (*) p<.10, ns p≥.10). Dots show individual measurements, bars indicate mean ± SD.

With both sexes pooled **(A)**, heterozygous animals started differing from wildtype animals in motor function at age 18 months. Analysing both sexes separately, we confirmed the effect of a motor phenotype present at age 18 months in the female group **(B)**, which might be mainly driving the observed overall group effect of the motor phenotype. There was also a trend towards an effect in males **(C)**, but larger group sizes per sex would be needed to draw more thorough conclusions on whether there is a sex-specific effect on the motor phenotype in this mouse model.

Appendix Figure S3. Quantitative assessment of Purkinje cell alterations and cell loss in the 304Q SCA3 mouse model.

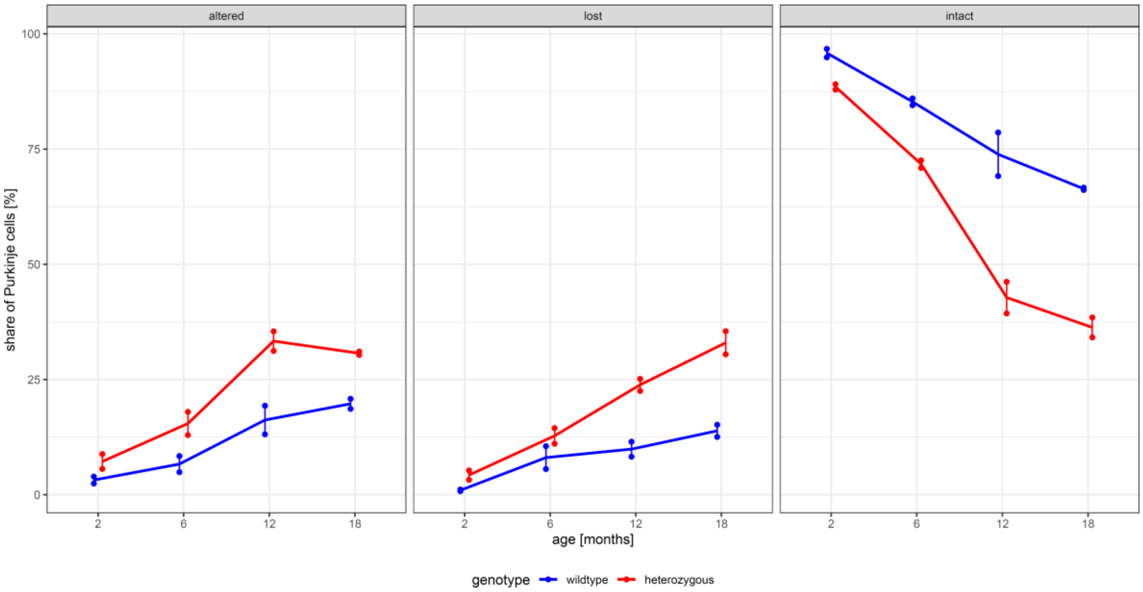

Purkinje cells of heterozygous SCA3 (red) and wildtype mice (blue) were manually counted and classified as altered (= shrunken soma), lost (= soma invisible) or intact (= intact soma), based on Nf and Nissl stainings. Values are displayed as mean and range.

Appendix Figure S4. Quantitative assessment of general atrophy in the 304Q SCA3 mouse model.

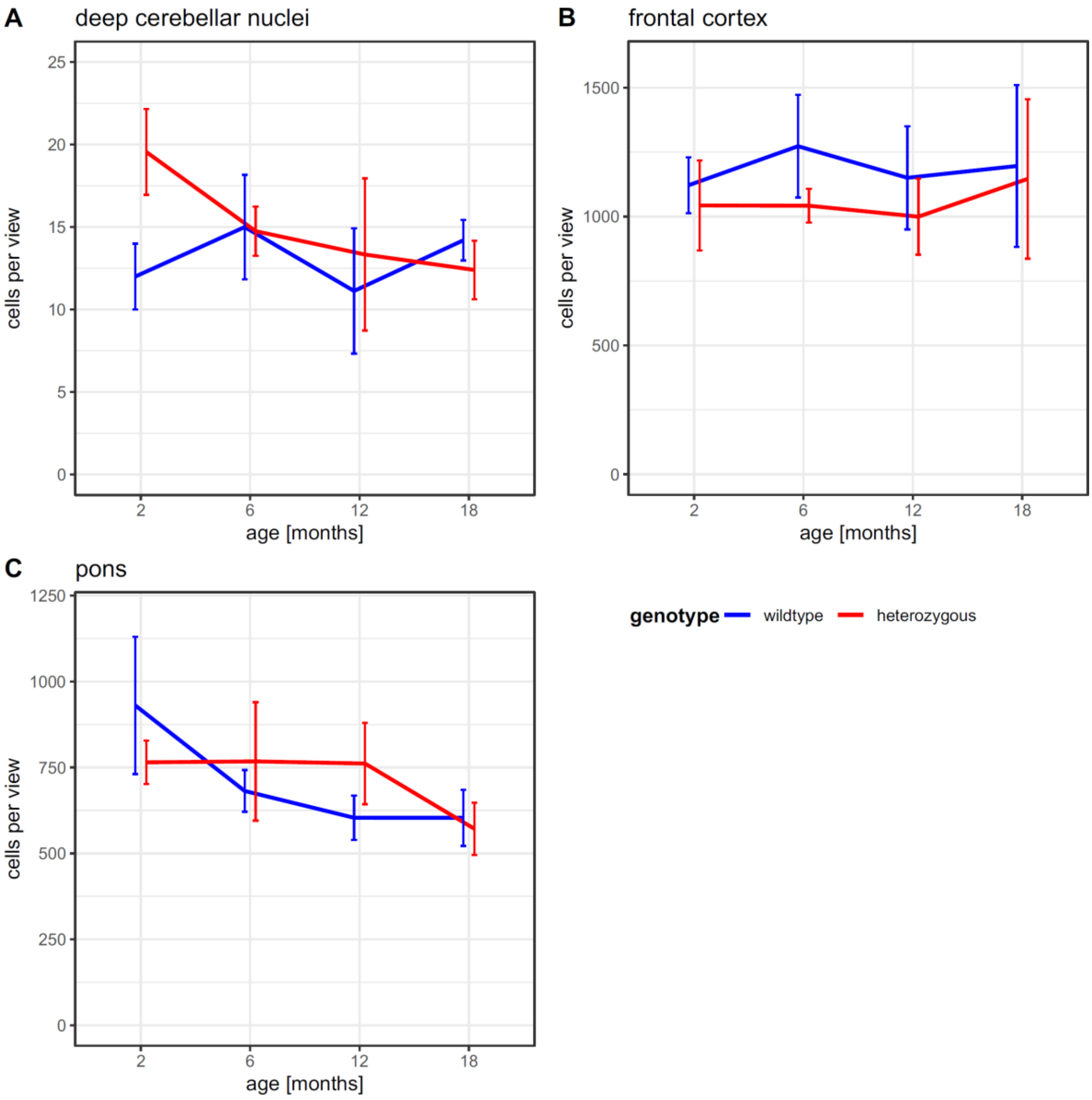

For heterozygous SCA3 (red) and wildtype mice (blue), cells per view were automatically counted for three regions (A: deep cerebellar nuclei, B: frontal cortex, C: pons). Values are displayed as mean and SD.

Appendix Supplementary References

Jacobi H, du Montcel ST, Bauer P, Giunti P, Cook A, Labrum R, Parkinson MH, Durr A, Brice A, Charles P *et al* (2015) Long-term disease progression in spinocerebellar ataxia types 1, 2, 3, and 6: a longitudinal cohort study. *Lancet neurology* 14: 1101-1108

Koeppen AH (2018) The Neuropathology of Spinocerebellar Ataxia Type 3/Machado-Joseph Disease. *Adv Exp Med Biol* 1049: 233-241

Kuhle J, Barro C, Andreasson U, Derfuss T, Lindberg R, Sandelius A, Liman V, Norgren N, Blennow K, Zetterberg H (2016) Comparison of three analytical platforms for quantification of the neurofilament light chain in blood samples: ELISA, electrochemiluminescence immunoassay and Simoa. *Clin Chem Lab Med* 54: 1655-1661

Paulson H (2012) Machado-Joseph disease/spinocerebellar ataxia type 3. *Handbook of clinical neurology* 103: 437-449

Paulson HL, Shakkottai VG, Clark HB, Orr HT (2017) Polyglutamine spinocerebellar ataxias - from genes to potential treatments. *Nature reviews Neuroscience* 18: 613-626

Wilke C, Pujol-Calderón F, Barro C, Stransky E, Blennow K, Michalak Z, Deuschle C, Jeromin A, Zetterberg H, Schüle R *et al*, 2019. Correlations between serum and CSF pNfH levels in ALS, FTD and controls: a comparison of three analytical approaches, Clinical Chemistry and Laboratory Medicine (CCLM).
