# Supplementary material for: Neurofilaments in spinocerebellar ataxia type 3: blood biomarkers at the preataxic and ataxic stage in humans and mice
Source: EMBO Mol Med. 2020 Jun 8;12(7):e11803. doi: 10.15252/emmm.201911803 (PMC7338806; doi:10.15252/emmm.201911803)
Supplement: Supplementary file 2 — Expanded View Figures PDF [file EMMM-12-e11803-s002.pdf]

Expanded View Figures

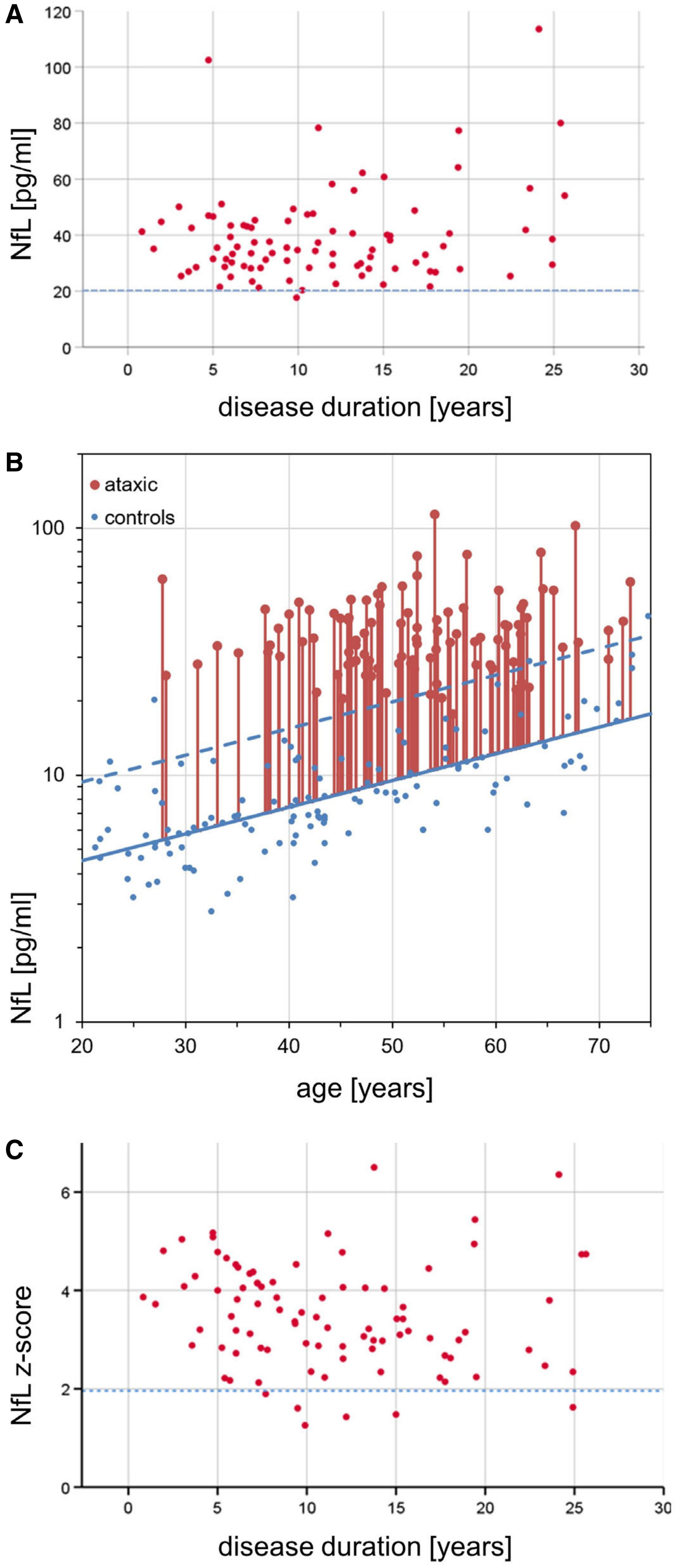

Figure EV1. Sustained increase of NfL levels through the ataxic stage.

A NfL levels of ataxic SCA3 subjects did not change significantly with disease duration, as indicated by linear regression using the pooled data of both cohorts ($F(1,90) = 1.56$, $P = 0.214$, $R^2 = 0.02$; slope: 1.37 [−0.81 to 3.55] [95% CI]). This finding demonstrates a sustained increased NfL release from degenerating neurons throughout the disease course of SCA3.

B To compare ataxic SCA3 subjects with controls at the same age, we expressed the measured NfL level of SCA3 subjects as NfL *z*-score in relation to the age-dependent NfL distribution in controls.

C The NfL *z*-score of ataxic SCA3 subjects did not change significantly with disease duration, as revealed by linear regression ($F(1,90) = 0.18$, $P = 0.670$, $R^2 < 0.01$; slope: −0.01 [−0.05 to 0.03], indicating that NfL level remained stably increased through the ataxic stage of SCA3. As NfL levels increase with disease severity and disease progression rate (see main text), but not with disease duration (as shown here), they seem to capture the *functional* stage of SCA3 disease, rather than the mere *calendrical* estimate of duration of the disease. In addition, disease duration is an inherently difficult measure in ataxic patients as it relies on subjective, retrospective estimates.

Source data are available online for this figure.

 © 2020 The Authors

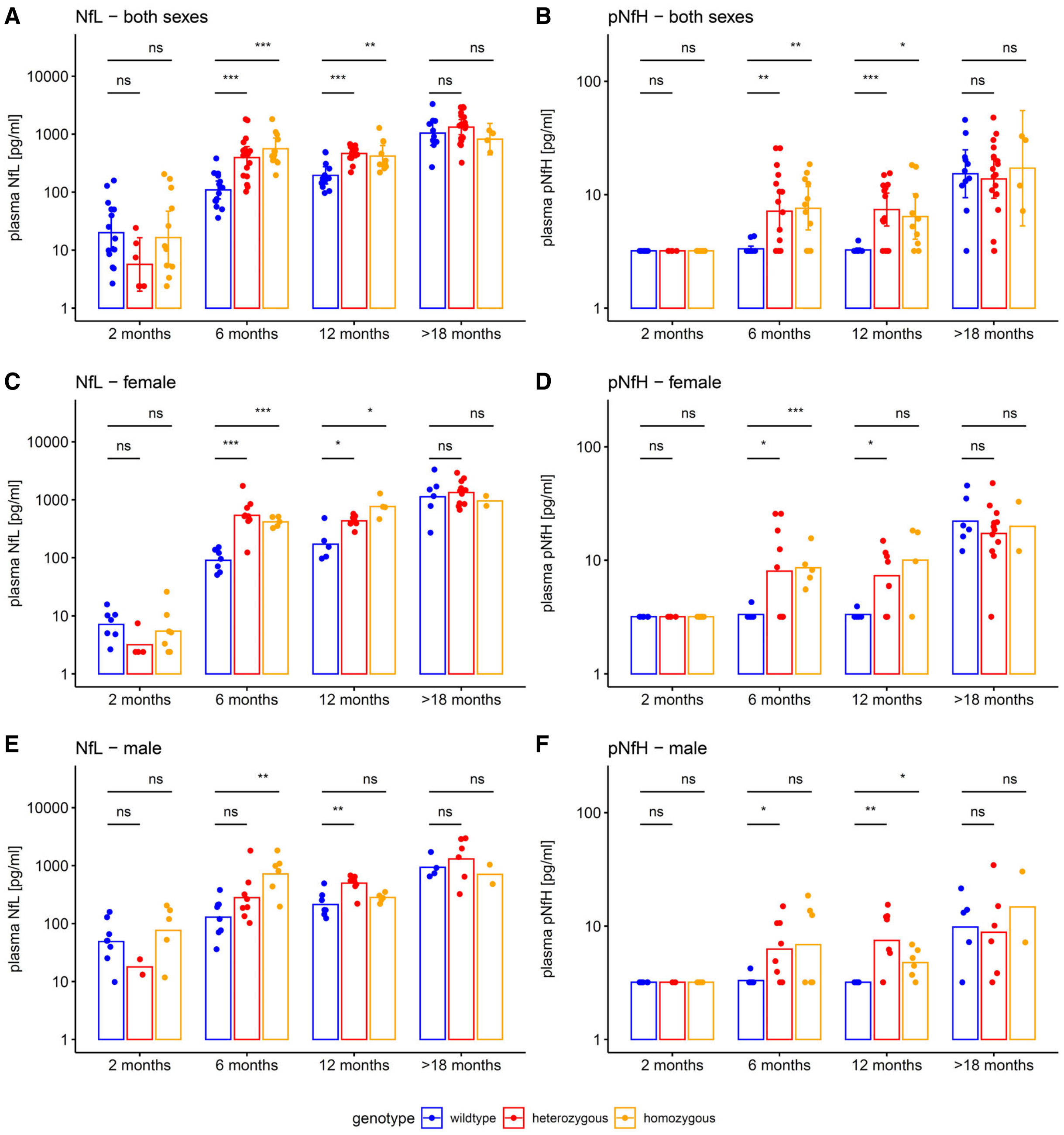

Figure EV2. Neurofilament plasma levels in heterozygous and homozygous SCA3 mice, including sex-specific analysis.

Plasma levels of NfL and pNfH were measured by Simoa assay in the SCA3 knock-in mouse model. Hetero- and homozygous animals were compared to wild-type animals by two-tailed unpaired *t*-tests, adjusted for unequal variances (***$P < 0.001$, **$P < 0.01$, *$P < 0.05$, ns $P \geq 0.05$, Bonferroni-corrected; see Appendix Table S3 for detailed statistics). Dots show individual measurements, bars indicate mean ± SD (calculated for log-transformed values).

A, C, E NfL levels. With both sexes pooled (A), heterozygous animals started differing from wild-type animals regarding NfL levels at age 6 months. Analysing both sexes separately, we confirmed the NfL increase at age 6 months in females (C), and, though less pronounced, also in males (E). Homozygous mice qualitatively confirmed the findings from heterozygous mice.

B, D, F pNfH levels. With both sexes pooled (B), heterozygous animals started differing from wild-type animals regarding pNfH levels at age 6 months. Analysing both sexes separately, we confirmed the pNfH increase at age 6 months in both females (D) and males (F). Homozygous mice again qualitatively confirmed the findings from heterozygous mice.

Source data are available online for this figure.

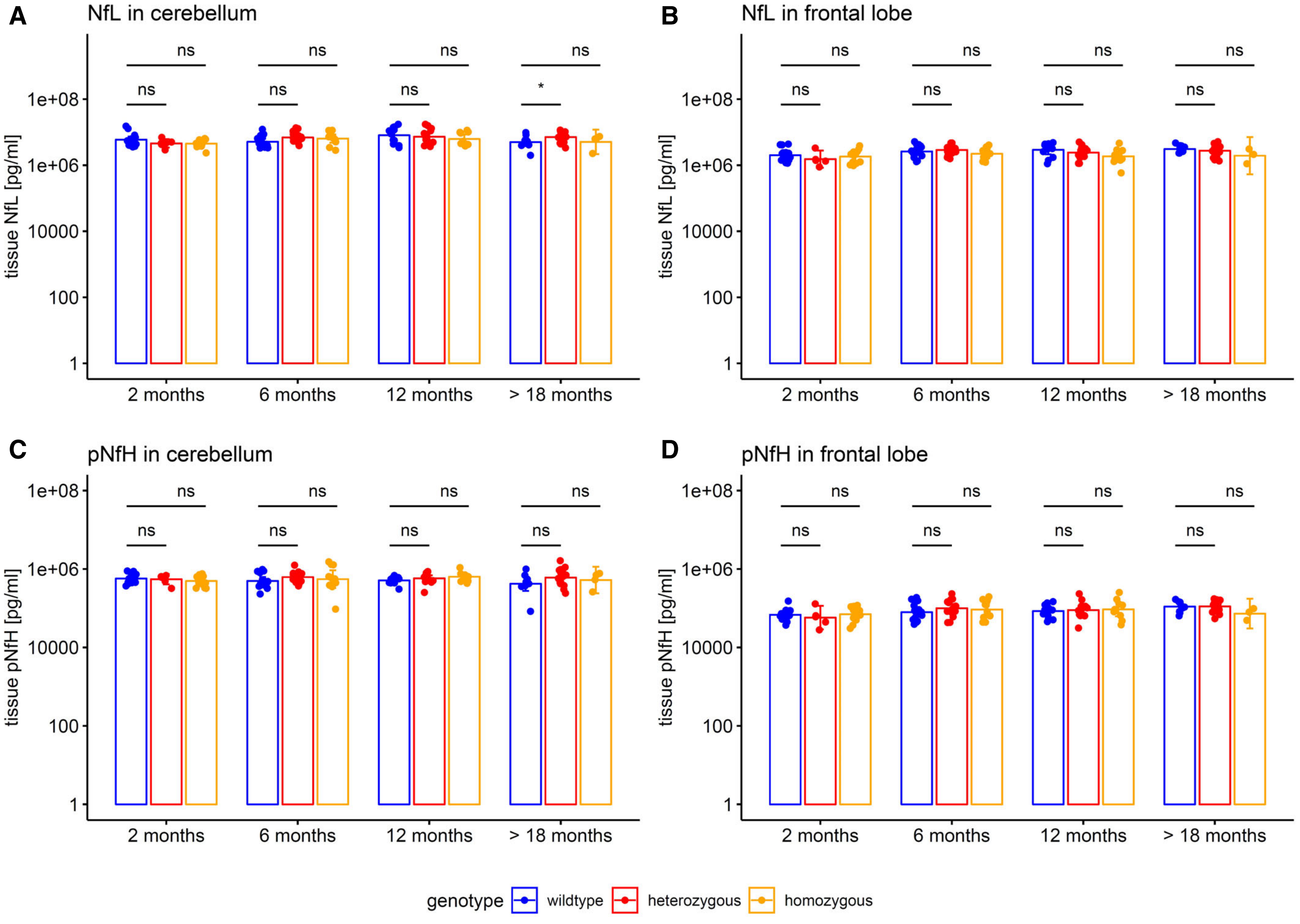

Figure EV3. Brain tissue levels of neurofilaments in murine SCA3 disease.

A–D Neurofilament tissue concentrations in the cerebellum (A, C) and the frontal lobe (B, D) were measured by Simoa assay in the SCA3 mouse model. Hetero- and homozygous animals were compared to wild-type animals by two-tailed unpaired *t*-tests, adjusted for unequal variances (*$P < 0.05$, ns $P \geq 0.05$, Bonferroni-corrected; see Appendix Table S3 for detailed statistics). Dots show individual measurements, bars indicate mean ± SD (calculated for log-transformed values).

Source data are available online for this figure.

© 2020 The Authors

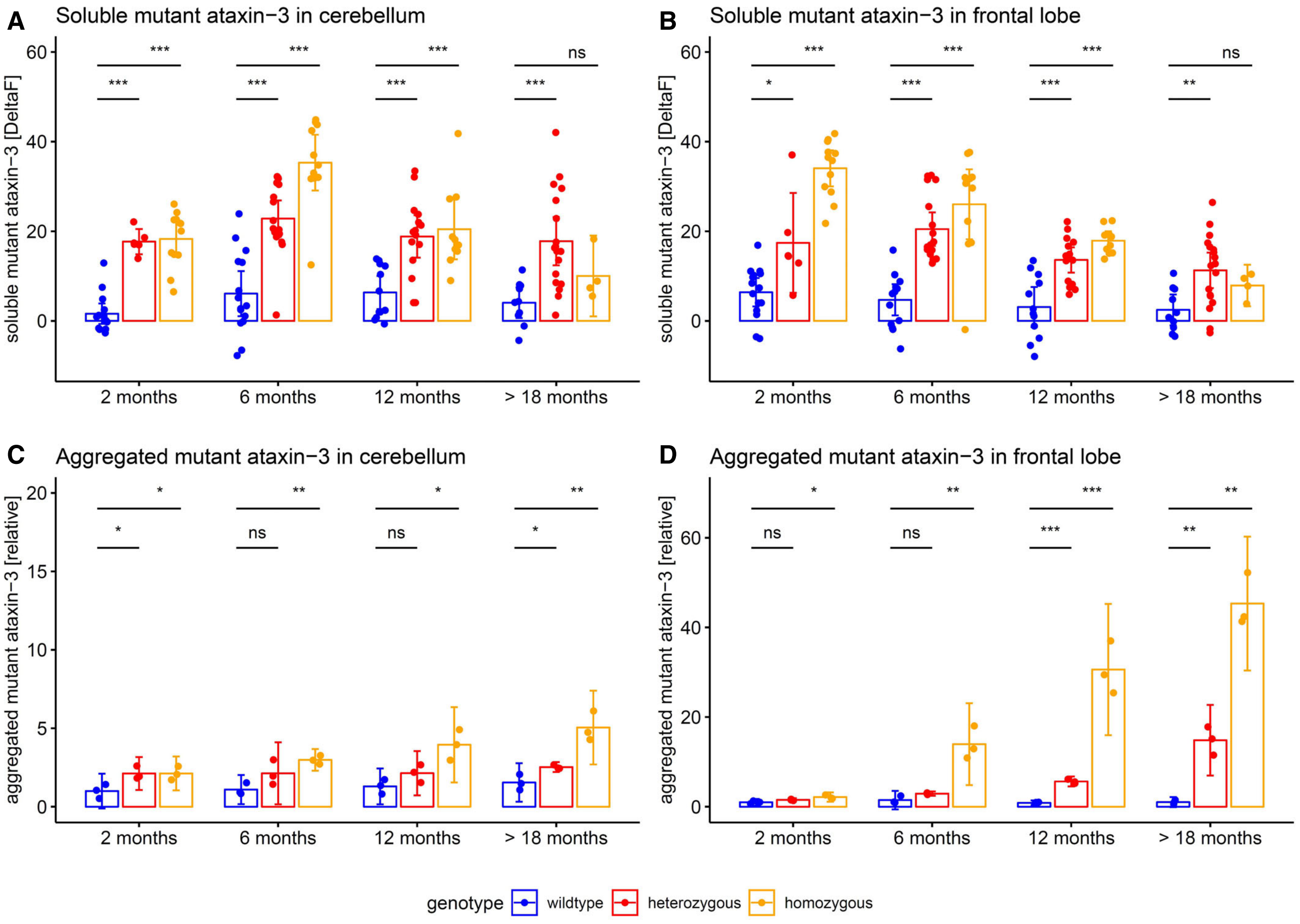

Figure EV4. Tissue levels of soluble and aggregated ataxin-3 in the cerebellum and frontal lobe in heterozygous and homozygous SCA3 mice.

A–D Tissue levels of soluble and aggregated mutant ataxin-3 were measured in cerebellum and frontal lobe. Hetero- and homozygous animals were compared to wild-type animals by two-tailed unpaired *t*-tests, adjusted for unequal variances (***$P < 0.001$, **$P < 0.01$, *$P < 0.05$, ns $P \geq 0.05$; see Appendix Table S3 for detailed statistics). Dots show individual measurements, bars indicate mean ± SD (calculated for log-transformed values). Homozygous mice confirmed the findings from heterozygous mice.

Source data are available online for this figure.

© 2020 The Authors

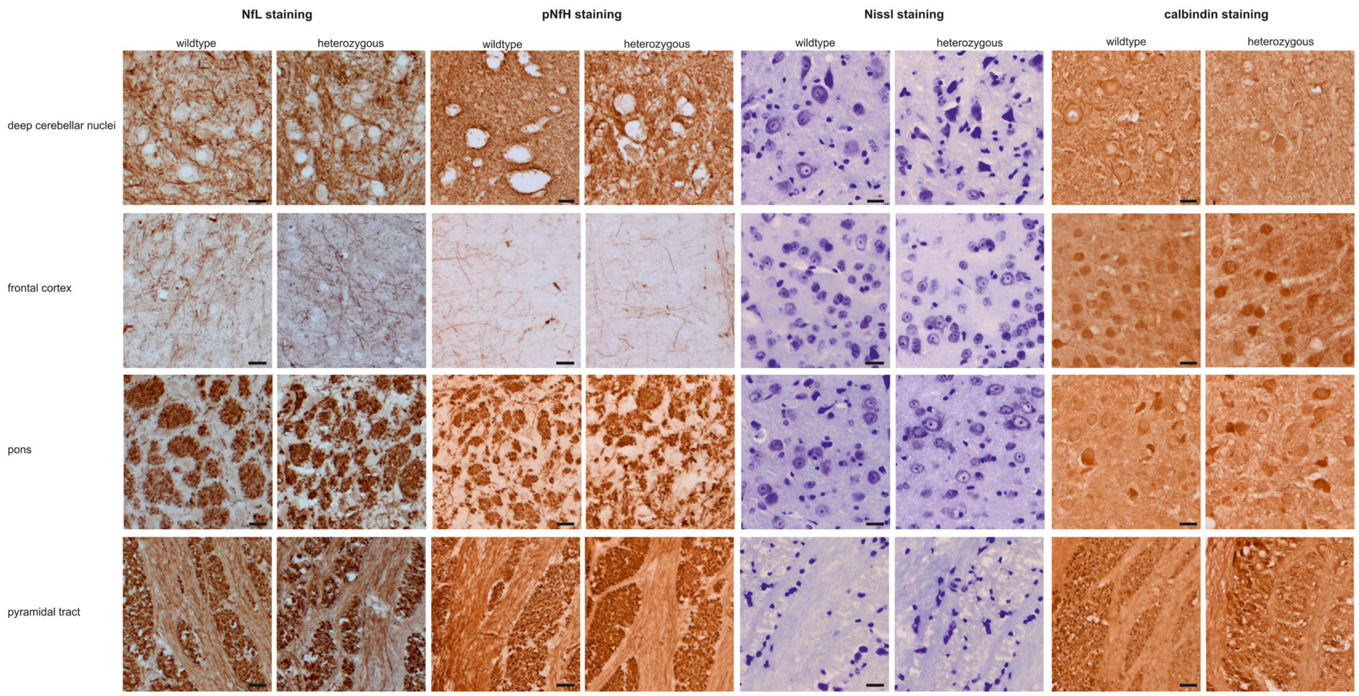

Figure EV5. Assessment of general atrophy in different brain regions of SCA3 mice.

We histologically assessed the extent of general atrophy in heterozygous SCA3 mice (using NfL, pNfH, Nissl and calbindin staining) in different brain regions (deep cerebellar nuclei, frontal cortex, pons and pyramidal tract), comparing heterozygous 304Q SCA3 mice at 2, 6, 12 and 18 months of age with wild-type animals. As these stainings did not show any marked differences between heterozygous and wild-type animals in overall cell count per view (as quantified from Nissl staining) at any age, we here present sections of 18-month-old animals in which any atrophy should be most pronounced. The sections of all other time points (2, 6, 12 months) are available in the source data. ML: molecular layer, PC: Purkinje cell, GL: granular layer, scale bars: 20 μm.

Source data are available online for this figure.

 © 2020 The Authors
